# Conventional Two-Stage Hepatectomy or Associating Liver Partitioning and Portal Vein Ligation for Staged Hepatectomy for Colorectal Liver Metastases? A Systematic Review and Meta-Analysis

**DOI:** 10.3389/fonc.2020.01391

**Published:** 2020-08-21

**Authors:** Liang Zhang, Zhentao Yang, Shiyu Zhang, Wenchao Wang, Shusen Zheng

**Affiliations:** ^1^Division of Hepatobiliary and Pancreatic Surgery, Department of Surgery, School of Medicine, The First Affiliated Hospital, Zhejiang University, Hangzhou, China; ^2^NHC Key Laboratory of Combined Multi-Organ Transplantation, Key Laboratory of the Diagnosis and Treatment of Organ Transplantation, CAMS, Hangzhou, China

**Keywords:** colorectal liver metastases, hepatectomy, associating liver partitioning and portal vein ligation for staged hepatectomy, two-stage hepatectomy, systematic review

## Abstract

**Background:** Pushing the surgical limits for initially unresectable colorectal liver metastases (CRLM) are two approaches for sequential liver resection: two-stage hepatectomy (TSH) and associating liver partitioning and portal vein ligation for staged hepatectomy (ALPPS). However, the role of each treatment modality remains ill-defined. The present meta-analysis was designed to compare the safety, efficacy, and oncological benefits between ALPPS and TSH in the management of advanced CRLM.

**Methods:** A systematic literature search was conducted from online databases through to February 2020. Single-arm synthesis and cumulative meta-analysis were performed.

**Results:** Eight studies were included, providing a total of 409 subjects for analysis (ALPPS: *N* = 161; TSH: *N* = 248). The completions of the second stage of the hepatectomy [98 vs. 78%, odds ratio (OR) 5.75, *p* < 0.001] and R0 resection (66 vs. 37%; OR 4.68; *p* < 0.001) were more frequent in patients receiving ALPPS than in those receiving TSH, and the waiting interval was dramatically shortened in ALPPS (11.6 vs. 45.7 days, weighted mean difference = −35.3 days, *p* < 0.001). Nevertheless, the rate of minor complications was significantly higher in ALPPS (59 vs. 18%, OR 6.5, *p* < 0.001) than in TSH. The two treatments were similar in 90-day mortality (7 vs. 5%, *p* = 0.43), major complications (29 vs. 22%, *p* = 0.08), posthepatectomy liver failure (PHLF; 9 vs. 9%, *p* = 0.3), biliary leakage (11 vs. 14%, *p* = 0.86), length of hospital stay (27.95 vs. 26.88 days, *p* = 0.8), 1-year overall survival (79 vs. 84%, *p* = 0.61), 1-year recurrence (49 vs. 39%, *p* = 0.32), and 1-year disease-free survival (34 vs. 39%, *p* = 0.66). Cumulative meta-analyses indicated chronological stability for the pooled effect sizes of resection rate, 90-day mortality, major complications, and PHLF.

**Conclusions:** Compared with TSH, ALPPS for advanced CRLM resulted in superior surgical efficacy with comparable perioperative mortality rate and short-term oncological outcomes, while this was at the cost of increased perioperative minor complications.

## Introduction

For patients with liver-limited metastases from colorectal cancer, radical surgery with complete resection of the metastases represents the most effective strategy, which could markedly improve prognosis and provide a potentially curative opportunity (1). However, the surgical procedure is a technological challenge in cases of insufficient future liver remnant (FLR), thereby preventing this subset of the population from undergoing surgical resection (2–4). Pushing this technological limit and expanding the pool of surgical candidates are two approaches for the sequential resection of metastases: two-stage hepatectomy (TSH) and associating liver partitioning and portal vein ligation for staged hepatectomy (ALPPS), thanks to the advent of preoperative liver volume modulation techniques (3). Patients with colorectal liver metastases (CRLM) that were historically deemed ineligible for liver resection due to inadequate FLR are now offered surgical approaches and, thus, the opportunity for complete tumor removal or even cure.

TSH typically consists of two separate stages of operations: the first stage includes complete tumor clearance of the FLR, contralateral portal vein embolization/ligation (PVE/PVL) to induce FLR hypertrophy, and, following a waiting period of ~4–8 weeks, a final extended hepatectomy in the second stage. This strategy, although well-established, has inherited risks of tumor progression between stages and insufficient volume gain of the FLR, leading to a dropout rate of ~30% (5, 6). It has also been reported that patients failing to proceed to the second stage of TSH had no significant survival benefits over those receiving chemotherapy only (7, 8). Subsequently, ALPPS was introduced as a novel technology incorporating liver parenchymal transection between the deportalized part and the FLR during the first stage (9). ALPPS was shown to allow much faster expansion of FLR volume over a dramatically reduced waiting interval (1–2 weeks), and resection rates (RRs) as high as 90–100% could be achieved. However, ALPPS was soon met with skepticism toward its technological safety, given its high mortality rate of up to 20% in initial reports with small numbers of patients (9, 10). Preliminary investigations of ALPPS also raised concerns regarding the risk of early postoperative tumor recurrence but also confirmed the feasibility of rescue ALPPS after the failure of PVE or PVL (11, 12).

Although several studies have been conducted to compare ALPPS with TSH for the management of advanced CRLM, the results greatly diverged. Some studies were in favor of TSH because ALPPS was found to show no advantages in achieving resectability while also resulting in higher morbidity rates, while others reported that ALPPS significantly improved resectability with similar frequencies of complications to TSH (13–15). It remains unclear which treatment modality may yield the best surgical results. A previous meta-analysis including nine retrospective studies showed that TSH for CRLM exhibited lower perioperative morbidity and mortality rates than ALPPS, suggesting the superiority of TSH (16). Nonetheless, the synthesis was heterogeneous in essence because patients who underwent liver resection for non-CRLM malignancies were also included in this meta-analysis (17, 18). Furthermore, the debate remains largely open regarding the learning curve effect for ALPPS. Indeed, a recent and so far the only multicenter randomized controlled trial (RCT) revealed that ALPPS and TSH for CRLM had comparable perioperative morbidity and mortality, yet the former achieved higher RRs (19).

With the inclusion of the latest surgical results and exclusion of heterogeneous indications, the present meta-analysis was designed to compare the safety, efficacy, and oncological benefits between ALPPS and conventional TSH for the management of patients with initially unresectable CRLM.

## Methods

### Search Strategy and Literature Selection

The review protocol was registered with the International Prospective Register of Systematic Reviews (PROSPERO) database (protocol number, CRD42020161596) (http://www.crd.york.ac.uk/PROSPERO/). We performed a systematic literature search for relevant studies using the PubMed, Web of Science, Embase, and Cochrane Library databases through to January 2020. The main medical terms used for the search included “colorectal liver metastasis,” “CRLM,” “CLM,” “liver resection,” “two-stage hepatectomy,” “TSH,” “associating liver partition and portal vein ligation for staged hepatectomy,” “*in situ* splitting,” and “ALPPS.” A detailed description of our online search strategy is provided in Supplementary Table 1. The reference lists of eligible studies were also manually searched to identify additional publications pertaining to our study. There were no restrictions on language or publication date.

The retrieved records were assessed according to the Preferred Reporting Items for Systematic Reviews and Meta-Analyses (PRISMA) statement (20). We included clinical studies reporting on a direct comparison of ALPPS vs. TSH in the treatment of CRLM. Excluded from the analysis were case reports, letters to the editor, commentary articles, conference abstracts, experimental assays, reviews, non-comparative studies, and studies including patients who underwent hepatectomies for non-CRLM malignancies.

### Study Outcomes, Data Extraction, and Quality Assessment

The primary outcomes of interest were perioperative morbidity and mortality, and surgical efficacy and oncological benefits served as the secondary outcomes. Data were collected from the eligible studies and mainly included baseline details, study characteristics, primary outcomes [90-day mortality, stage 1/stage 2/overall major complications (MaCs), stage 1/stage 2/overall minor complications (MiCs)], and secondary outcomes [RR, R0 rate, amount of estimated blood loss, total length of hospital stay, waiting interval, FLR volume, FLR/total liver volume (TLV) ratio, 1-year overall survival (OS) rate, 1-year recurrence rate, and 1-year disease-free survival (DFS) rate]. We contacted the corresponding authors of the included studies for missing information when needed. MaCs were defined as any perioperative complications of grade III or higher according the Dindo-Clavien classification system, and MiCs were defined as those of below grade III. Calculations of the proportions of patients developing stage 2 complications were based on patients who were successfully surgically treated (per protocol), while other outcome measurements were based on the intention-to-treat population.

The Cochrane Handbook for Systematic Reviews of Interventions (version 5.1.0) was employed for quality evaluation of the included RCTs, and the modified Newcastle-Ottawa Quality Assessment Scale was used to assess the risk of bias of observational studies (21, 22). A star system of quality scores ranging from 0 to 9 was applied for each included study, and those scoring eight stars or more were considered high quality. Two independent reviewers (ZTY and SYZ) were responsible for the data extraction and literature quality assessment. Disagreements were resolved by consensus, and then confirmed by another reviewer (WCW).

### Statistical Analysis

For pooled analysis, the odds ratio (OR) with 95% confidence intervals (CIs) was calculated for dichotomous variables, and the weighted mean difference (WMD) with 95% CIs was estimated for quantitative variables. A single-arm meta-analysis was conducted to obtain the absolute summary estimate values of each study outcome. In addition, a cumulative meta-analysis was performed to evaluate the chronological stability of the pooled effect sizes when there were five or more studies reporting data on the same outcome variable (23, 24). Heterogeneity among the studies was assessed using the Cochran chi-squared test and *I*^2^, where *I*^2^ >50% suggested significant heterogeneity. Galbraith radials and L'Abbe plot were also used to visually evaluate the extent of heterogeneity and investigate the potential sources of heterogeneity. A random effects model was used to pool the data when I^2^ >50%, while a fixed effects model was applied when I^2^ ≤ 50%. Sensitivity analysis was performed to assess the impact of excluding individual studies on the pooled results. Funnel plot analysis and Begg's and Egger's tests were used to detect the publication bias. *P* ≤ 0.05 for the Begg's test and *P* ≤ 0.05 for Egger's test were quantitatively indicative of an obvious publication bias. Statistical analysis was carried out using STATA version 12.0 (Stata Corporation, College Station, TX). A two-tailed *P-*value of 0.05 or less was considered to be statistically significant.

## Results

### Study Selection and Baseline Characteristics

**Figure 1:**
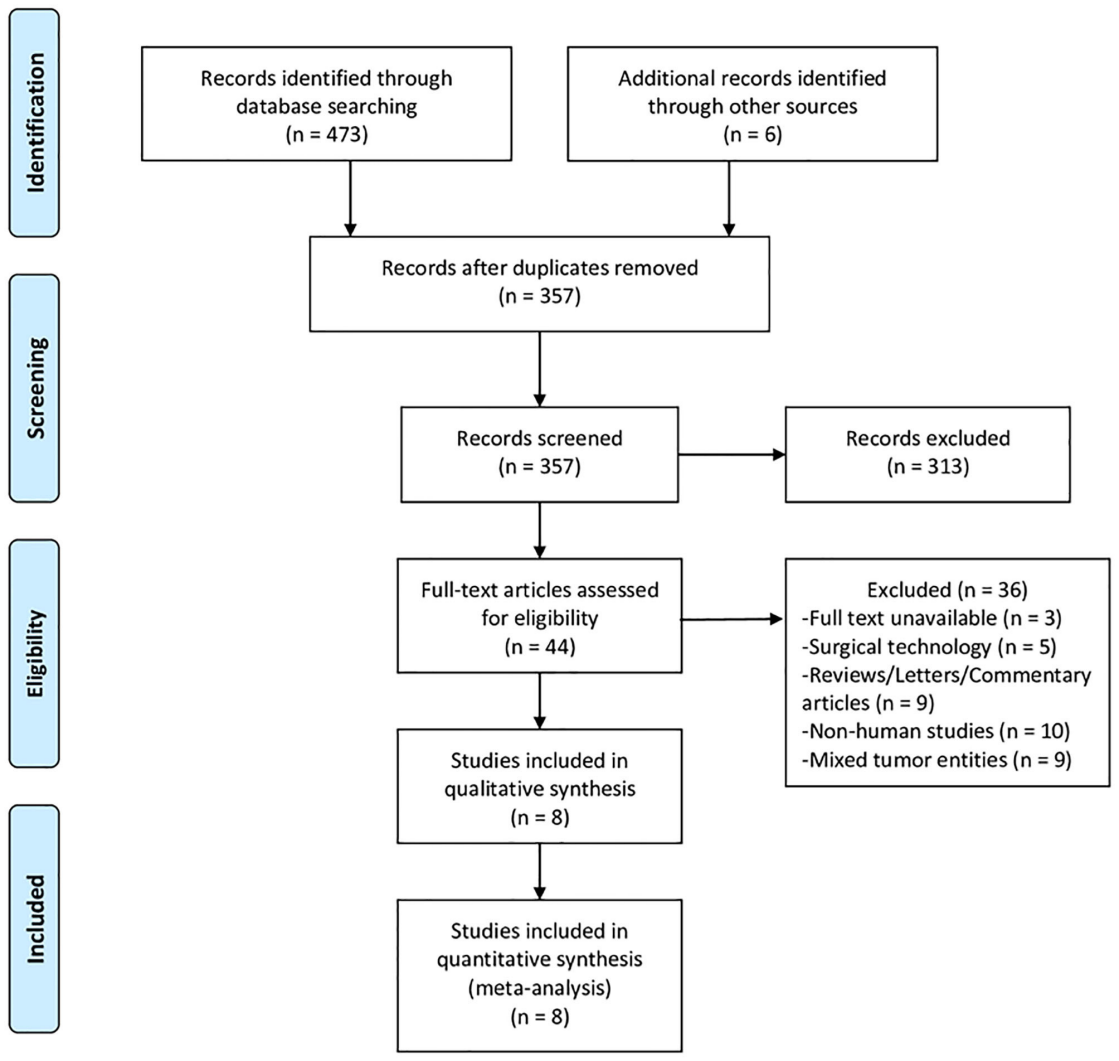
PRISMA flow diagram depicting the selection process of studies included for meta-analysis.

The online database search and manual survey yielded a total of 479 citations. The selection processes are depicted in Figure 1, and eventually, eight clinical studies were included for this meta-analysis (13–15, 19, 25–28). In one of these studies, TSH was compared with Tourniquet-ALPPS, a technological modification of ALPPS that uses a tourniquet to occlude the intrahepatic collaterals. Another study (28) had overlapping cohorts with a previous multicenter RCT (19) and was subjected to the quantitative synthesis only when reporting unique endpoints. The baseline characteristics of the included studies are displayed in Table 1, and the results of the literature quality evaluation are shown in Supplementary Figure 1.

**Table 1:** Characteristics of the included studies.

**References**	**Country**	**Patients**	**Interventions**	**Observation period**	**Study design**
Robles-Campos et al. (13)^*^	Spain	CRLM	ALPPS vs. TSH	2000–2016	Retrospective, PSM
Baumgart et al. (27)	Germany			2008–2017	Retrospective
Sandstrom et al. (19)	Danish, Norwegian, Swedish			2014–2016	RCT
Rosok et al. (28)^#^	Norwegian			2014–2016	RCT
Kikuchi et al. (26)	Japan			Up to April 2015	Retrospective
Kambakamba et al. (25)	Switzerland			2002–2015	Retrospective
Adam et al. (14)	France			2010–2014	Retrospective
Ratti et al. (15)	Italy			2008–2013	Retrospective, PSM

* *Tourniquet-ALPPS was adopted in this study, which is a technical modification of ALPPS by placing a tourniquet on the hepatic bipartition line during stage 1*.

# *Fourty-three patients enrolled in the multicenter study by Sandstrom et al. underwent surgeries at one of the six involved hospitals, and twenty-four of them were included in the substudy by Rosok et al*.

*ALPPS, associating liver partitioning and portal vein ligation for staged hepatectomy; CRLM, colorectal liver metastases; PSM, propensity score matching; RCT, randomized controlled trial; TSH, two-stage hepatectomy*.

The analysis comprised a total of 409 patients with CRLM, of whom 161 underwent ALPPS and the remaining 248 received TSH. Patient characteristics in each included study, stratified by treatment modality, are summarized in Supplementary Table 2. Comparisons of demographic characteristics between ALPPS and TSH groups are displayed in Supplementary Table 3. Compared with patients who received TSH, those who underwent ALPPS were older (62.78 vs. 65.86 years, *p* < 0.001). Otherwise, the two groups were comparable in regard to gender, American Society of Anesthesiology scores, primary tumor location, synchronous or metachronous metastasis, presence of extrahepatic disease, preoperative carcinoembryonic antigen level, number of liver lesions, and mean tumor size, as well as the administration and mean cycle of neoadjuvant chemotherapy.

### Meta-Analysis: ALPPS vs. TSH

The pooled results of the primary analyses are exhibited in Table 2. ALPPS and TSH had comparable 90-day mortality rates (7 vs. 5%, *p* = 0.43-, rates of MaCs (stage 1 MaCs: 7 vs. 8%, *p* = 0.65; stage 2 MaCs: 22 vs. 23%, *p* = 0.99; overall MaCs: 29 vs. 22%, *p* = 0.08), total length of hospital stay (27.95 vs. 26.88 days, *p* = 0.8), estimated blood loss during the first stage (643.95 vs. 263.27 ml, *p* = 0.07), 1-year OS rate (79 vs. 84%, *p* = 0.61), 1-year tumor recurrence rate (49 vs. 39%, *p* = 0.32) and 1-year DFS rate (34 vs. 39%, *p* = 0.66). ALPPS had significantly higher RRs (98 vs. 78%, OR 5.75, *p* < 0.001) and R0 rates (66 vs. 37%, OR 4.68, *p* < 0.001) than TSH, as well as a shortened waiting interval (11.6 vs. 45.7 days, WMD= −35.3 days, *p* < 0.001) and reduced blood loss during the second stage (316.42 vs. 1,046.5 ml, WMD = −742.66 ml, *p* < 0.001). Despite similar preoperative FLR volumes (WMD = 5.48 ml, *p* = 0.73) and FLR/TLV ratios (WMD = 1.09%, *p* = 0.24), ALPPS had a significantly greater FLR volume (WMD = 141.47 ml, *p* < 0.001) and FLR/TLV ratio (WMD = 10.93%, *p* < 0.001) than TSH at 1 week after the first interventions. Nonetheless, ALPPS patients were at significantly increased risks of developing MiCs when compared to TSH patients (stage 1 MiCs: 52 vs. 10%, *p* < 0.001; stage 2 MiCs: 53 vs. 25%, *p* = 0.004; overall MiCs: 59 vs. 18%, *p* < 0.001). The cumulative meta-analysis confirmed chronological stability for the effect sizes of 90-day mortality, RRs and overall MaCs.

**Table 2:** Meta-analysis of ALPPS vs. TSH group.

**Outcomes of interests**	**No. of studies**	**No. of patients**	**Single armed synthesis**		**OR/WMD [95% CI]**	***p*-value**	**Heterogeneity**	
			**ALPPS group [95% CI]**	**TSH group [95% CI]**			**I^**2**^ %**	***P*-value**
90-day mortality	7	409	0.07 [0.03, 0.12]	0.05 [0.02, 0.08]	1.39 [0.62, 3.14]	0.43	0	0.90
Stage 1 MaCs	3	148	0.07 [0.00, 0.24]	0.08 [0.01, 0.18]	1.31 [0.41, 4.19]	0.65	0	0.95
Stage 2 MaCs	3	129	0.22 [0.07, 0.42]	0.23 [0.14, 0.34]	1.01 [0.28, 3.62]	0.99	50.5	0.13
Overall MaCs	5	319	0.29 [0.17, 0.43]	0.22 [0.14, 0.32]	1.60 [0.94, 2.71]	0.08	0	0.58
Stage 1 MiCs	3	148	0.52 [0.2, 0.84]	0.1 [0.04, 0.17]	13.58 [5.02, 36.76]	<0.001	0	0.71
Stage 2 MiCs	3	129	0.53 [0.28, 0.77]	0.25 [0.16, 0.36]	2.63 [1.21, 5.69]	0.004	0	0.37
Overall MiCs	2	106	0.59 [0.4, 0.77]	0.18 [0.1, 0.28]	6.5 [2.52, 16.71]	<0.001	0	0.58
RR	6	335	0.98 [0.93, 1.00]	0.78 [0.72, 0.83]	5.57 [2.45, 12.69]	<0.001	14.4	0.32
R0 rate	3	209	0.66 [0.14, 1.00]	0.37 [0.08, 0.72]	4.68 [2.23, 9.84]	<0.001	49.9	0.14
Stage 1 blood loss (ml)	2	129	643.95 [461.57, 826.32]	263.27 [25.93, 500.61]	393.68 [−30.74, 818.1]	0.07	94.5	<0.001
Stage 2 blood loss (ml)	2	129	316.42 [286.96, 345.89]	1046.5 [960.5, 1132.57]	−742.66 [−835.45, −649.87]	<0.001	0	0.76
Length of hospital stay (d)	2	129	27.95 [18.25, 37.65]	26.88 [9.63, 44.13]	0.97 [−6.57, 8.51]	0.8	70.9	0.06
Waiting interval (d)	2	129	11.6 [11.30, 11.89]	45.7 [41.00, 50.35]	−35.3 [−37.72, −32.88]	<0.001	48.7	0.16
Preoperative FLR (ml)	2	129	338.22 [284.59, 391.86]	326.8 [251.34, 402.25]	5.48 [-25.02, 35.98]	0.73	0	0.5
Preoperative FLR/TLV (%)	2	129	27.71 [16.75,38.67]	27.35 [15.10, 39.6]	1.09 [−0.70, 2.89]	0.24	0	0.69
Interstage FLR (ml)^*^	2	129	539.9 [406.76, 673.07]	403.5 [311.42, 495.64]	141.47 [95.59, 187.34]	<0.001	0	0.4
Interstage FLR/TLV (%)^*^	2	129	41.2 [33.90, 49.55]	30.38 [21.75, 39.00]	10.93 [8.47, 13.39]	<0.001	0	0.92
1-year OS rate	3	122	0.79 [0.65, 0.90]	0.84 [0.66, 0.96]	0.78 [0.29, 2.07]	0.61	0	0.98
1-year recurrence rate	3	124	0.49 [0.35, 0.63]	0.39 [0.28, 0.51]	1.46 [0.69, 3.08]	0.32	0	0.68
1-year DFS rate	4	201	0.34 [0.05, 0.70]	0.39 [0.15, 0.67]	0.85 [0.42, 1.73]	0.66	40.3	0.17

* *At 1 weeks after the first interventions*.

*ALPPS, associating liver partitioning and portal vein ligation for staged hepatectomy; CI, confidence interval; d, day(s); DFS, disease-free survival; FLR, future liver remnant; MaCs, major complications; MiCs, minor complications; OR, odds ratio; OS, overall survival; RR, resection rate; PHLF, post-hepatectomy liver failure; TLV, total liver volume; TSH, two-stage hepatectomy; WMD, weighted mean difference*.

### Subgroup Analysis

The primary analyses were repeated in a subgroup of high-quality studies. Six studies (13–15, 19, 27, 28) were considered high quality according to the results of the literature quality evaluation and were subjected to subgroup analysis. As depicted in Table 3-1, the summary estimates in this subset showed consistency when compared with those of the holistic analysis.

**Table 3:** Results of subgroup analyses.

**Outcomes of interest**	**No. of studies**	**No. of patients**	**Single armed synthesis**		**OR [95% CI], *p-*value**	**Heterogeneity (I^**2**^%, *p*-value)**
			**ALPPS group [95% CI]**	**TSH group [95% CI]**		
Table 3-1. Subgroup analysis of high quality studies^*^						
90-day mortality	5	303	0.05 [0.01, 0.11]	0.05 [0.02, 0.09]	1.21 [0.45, 3.25], 0.71	0, 0.72
Overall MiCs	3	203	0.47 [0.26, 0.67]	0.19 [0.12, 0.26]	3.78 [1.43, 10.00], 0.007	50.1, 0.14
Overall MaCs	4	245	0.32 [0.16, 0.49]	0.24 [0.14, 0.35]	1.67 [0.92, 2.99], 0.09	0, 0.43
Resection rate	5	303	0.98 [0.94, 1.00]	0.78 [0.62, 0.91]	9.51 [3.86, 23.47], <0.001	0, 0.55
1-year OS rate	2	90	0.82 [0.67, 0.94]	0.9 [0.81, 0.97]	0.73 [0.2, 2.6], 0.62	0, 0.92
**Table 3-2. Subgroup analysis of PHLF and biliary leakage**						
Overall PHLF	6	312	0.09 [0, 0.24]	0.09 [0.05, 0.13]	1.50 [0.70, 3.24], 0.3	0, 0.58
Stage 1 PHLF	5	238	0.01 [0, 0.1]	0.01 [0, 0.04]	2.24 [0.54, 9.22], 0.26	34.5%, 0.22
Stage 2 PHLF	5	209	0.04 [0, 0.11]	0.09 [0.05, 0.15]	0.79 [0.28, 2.22], 0.67	0, 0.97
Overall biliary leakage	3	164	0.11 [0, 0.32]	0.14 [0.07, 0.21]	1.09 [0.42, 2.86], 0.86	41, 0.18
Stage 1 biliary leakage	4	196	0.01 [0, 0.07]	0.01 [0, 0.05]	2.74 [0.45,16.76], 0.28	0, 0.41
Stage 2 biliary leakage	4	164	0.19 [0.01, 0.48]	0.17 [0.10, 0.24]	1.54 [0.66,3.56], 0.32	0, 0.47

* *Only are the pooled results for which studies included for the synthesis changed during the subgroup analysis displayed*.

*ALPPS, associating liver partitioning and portal vein ligation for staged hepatectomy; CI, confidence interval; MaCs, major complications; MiCs, minor complications; OR, odds ratio; OS, overall survival; PHLF, post-hepatectomy liver failure; TSH, two-stage hepatectomy*.

In addition, posthepatectomy liver failure (PHLF) and biliary leakage, two major causes of mortality following extensive hepatectomy, were analyzed. The pooled results revealed no significant differences in PHLF and biliary leakage between ALPPS and TSH (Table 3-2). The cumulative meta-analysis suggested that the effect sizes of stage 2 PHLF and overall PHLF have stabilized between two groups with the gradually narrowed CIs.

### Heterogeneity, Sensitivity Analysis, and Publication Bias

Forest plots, L'Abbe plots, Galbraith plots, funnel plots, and sensitivity analysis of each outcome measures are displayed in Supplementary Figures 2–14. The heterogeneity of most study outcomes was acceptable, and the sensitivity analyses showed that the summary estimates changed quite mildly after excluding individual study, suggesting robustness of the results. The funnel plots indicated that the distribution of the included studies was symmetric, and Begg's and Egger's tests suggested no potential publication bias.

## Discussion

TSH and ALPPS have both expanded the surgical armamentarium for patients with initially unresectable CRLM due to inadequate FLR, and intriguingly, this disease entity has evolved as the leading indication for these two procedures (8, 29, 30). Although ALPPS used to be adopted for patients with primary hepatobiliary tumors requiring extensive liver resection in earlier stages, several subsequent series analyses reported unsatisfactory perioperative results in this population, necessitating a reevaluation of the surgical indications (31, 32). It becomes clear that CRLM had the lowest risks of complication and mortality after ALPPS, which was explained as a consequence of the favorable tumor biology and normal liver (33–35). Over the past few years, several meta-analyses have been conducted to compare ALPPS with TSH or other traditional FLR augmentation strategies but mainly for mixed indications (Supplementary Table 4) (16, 30, 36–42). The present meta-analysis laid special emphasis on the treatment of CRLM. In addition, we conducted single-arm meta-synthesis to derive the absolute values of the outcome variables and cumulative meta-analysis to assess the chronological stability of the pooled effect size. As two major adverse events after extended hepatectomy, PHLF and biliary leakage were also separately analyzed from the remaining complications. The results of our analysis confirmed that ALPPS for CRLM had superior surgical efficacy relative to TSH regarding its enhanced capability to induce FLR hypertrophy and achieve resectability. This advantage, however, was established at the cost of increased risks of MiCs. Importantly, ALPPS and TSH were comparable in terms of perioperative mortality, MaCs, PHLF and biliary leakage, indicating that the safety profiles of ALPPS were not severely compromised. The short-term oncological benefits were also similar between two groups. Our study updates the current knowledge on the role of ALPPS in the treatment of advanced CRLM and may thereby influence future treatment options.

The most important concern raised by hepatobiliary surgeons for ALPPS is probably its technology safety. Notably, a preliminary single-center study with 15 cases of ALPPS reported mortality and morbidity rates as high as 28.7 and 66.7%, respectively (10). Later, in a multicenter retrospective study, 25 out of 62 (40.3%) patients receiving ALPPS developed MaCs, and eight (12.9%) succumbed (43). Initially reported high morbidity and mortality rates of ALPPS were far beyond the accepted standard of liver surgery and labeled this procedure as extremely risky. In spite of the consensus that conventional hepatectomy for CRLM should have a perioperative mortality rate of <5%, much less have been actually reported about the mortality rate of complex hepatectomy, which may stand within the vicinity of 8% (44). It remains challenging to define an acceptable mortality rate for ALPPS that would comply with current surgical practice because patients who receive ALPPS are mostly at the margins of resectability and difficult to match.

Furthermore, the raw figures obtained from the preliminary reports needed to be interpreted cautiously in light of the learning effect of a novel surgical procedure. Although the early scenario was dismal, continuous improvements in the safety profiles of ALPPS have been witnessed over time (45). A retrospective report of 320 patients from the ALPPS registry, in which 72% were diagnosed with CRLM, documented a 90-day mortality of 8.8%, a rate which had come close to that reported after traditional major hepatectomy (46). More interestingly, after cutting out the effect of the learning curve by analyzing experienced centers only, a continuous drop in the perioperative mortality and morbidity of ALPPS was observed over time, as the mortality rates decreased from 17 to 4%, and major interstage complications, from 10 to 3% (33).

The gratifying gain in surgical safety of ALPPS was achieved mainly with sharpened patient selection and refinements in surgical procedures. Evolving efforts have been made to identify unfavorable clinical precondition factors to optimize patient selection and prevent the development of futile outcomes following ALPPS. Recently, the surgical indications of ALPPS have shifted toward younger CRLM patients. A single-center, prospective pilot study with meticulous selection of patients with CRLM reported zero perioperative mortality following ALPPS with a MaCs rate of 14% (47). Over the past decade, ALPPS had undergone manifold technical refinements to capitalize upon its advantages and improve its safety profiles. Although it is of paramount importance to assess the efficacy and safety of these refined ALPPS techniques in CRLM, there is lack of standardization of its technical variants, which therefore may not permit meaningful statistical comparisons (48). During the literature search, we found only one study that reported on a direct comparison between a modified ALPPS (Tourniquet-ALPPS) and TSH for CRLM (13). This study, included in the current analysis, demonstrated that the two treatments had similar rates of perioperative morbidity and mortality.

With the inclusion of the latest surgical results from 8 clinical studies involving 409 patients, our study yielded a 90-day mortality rate of 7% (95% CI, 0.03–0.12) after ALPPS, which was comparable with that of 5% (95% CI, 0.02–0.08) following TSH (OR, 1.39; 95% CI, 0.62–3.14; *p* = 0.43). The pooled effect sizes also showed chronological stability in the cumulative meta-analysis. These figures, although generated from the pooling of comparative studies, are in line with those obtained from non-comparative studies. A recent report of 486 patients with CRLM from the ALPPS registry documented a 90-day mortality rate of 7% (49). Meanwhile, previously reported perioperative mortality rates for patients who underwent TSH for CRLM ranged from 3.4 to 11.3%, with a peak in the distribution at ~6% (6, 7, 50, 51).

Although the perioperative mortality of ALPPS may approach a level similar to that of TSH, it had to be acknowledged that ALPPS still displays a greater frequency of complications. In the present study, perioperative complications were further stratified into MaCs and MiCs, as well as into stage 1 and stage 2, to better gauge the degree of severity and two-stage patterns. As the result, we found that ALPPS had a similar rate of MaCs (stage 1 MaCs: 7 vs. 8%, *p* = 0.65; stage 2 MaCs: 22 vs. 23%, *p* = 0.99; overall MaCs: 29 vs. 22%, *p* = 0.08) as TSH but a significantly higher rate of MiCs (stage 1 MiCs: 52 vs. 10%, OR 13.58, *p* < 0.001; stage 2 MiCs: 53 vs. 25%, OR 2.63, *p* = 0.004; overall MiCs: 59 vs. 18%, OR 6.5, *p* < 0.001). Our results suggest that the high morbidity rate in ALPPS was most likely attributable to an increased risk of MiCs. Future strategies are required to minimize the surgical invasiveness of ALPPS and to lower the risks of perioperative complications before ALPPS could achieve similar or even better safety profiles than traditional TSH.

The current study showed that ALPPS outperformed conventional TSH in terms of induction of remnant liver regeneration and, more importantly, achieved higher RRs (98 vs. 78%, OR 5.57, *p* < 0.001) and R0 rates (66 vs. 37%, OR 4.68, *p* < 0.001). Our findings are highly consistent with the existing evidence and once again affirmed the superiority of ALPPS in terms of surgical efficiency (18, 52, 53). This prominent advantage of ALPPS is precisely what instigated the initial enthusiasm in this novel surgical procedure. During ALPPS, the provision of a rapid increase in FLR dramatically shortens the waiting intervals and, more importantly, facilitated surgeons to proceed with the staged operation before the formation of adhesions or the threat of tumor progression. Supported by this notion, we found that ALPPS had a significantly shorter interstage interval (11.6 vs. 45.7 days, WMD= −35.3 days, *p* < 0.001) and reduced smaller blood loss during the second stage (316.42 vs. 1,046.5 ml, WMD= −742.66 ml, *p* < 0.001) relative to TSH.

The underlying mechanisms responsible for this strong hypertrophic stimulus remain largely unclear, but some valuable insights could be gained from the circulatory cytokine profiles. Experimental assays have demonstrated that compared with those receiving liver transection alone or PVL, mice undergoing ALPPS showed significantly accelerated liver hypertrophy relative to those receiving either liver transection alone or PVL, indicating a potentially less pronounced role of microcirculation discontinuity (52, 54). The gene expressions of promitogenic cytokines in regenerating the FLR and serum IL-6 levels were significantly increased in ALPPS-treated mice, with analogous results in human. More interestingly, the injection of plasma obtained from ALPPS-treated mice to PVL-treated mice, which omitted *in situ* transection, could even mimic a comparable degree of liver regeneration as in original ALPPS.

Despite the rapid volume increase of the FLR in ALPPS, we did not observe a congruent reduction in the risk of PHLF. In the present study, the proportions of patients developing PHLF were similar between ALPPS and TSH (stage 1 PHLF: 1 vs. 1%, OR 2.24, *p* = 0.26; stage 2 PHLF: 4 vs. 9%, OR 0.79, *p* = 0.67; overall PHLF: 9 vs. 9%, OR 1.5, *p* = 0.3). Consistently, several investigations suggested that ALPPS resulted in unprecedented growth of FLR volume but did not reduce the incidence of PHLF as substantially as expected, with PHLF still accounting for ~75% of ALPPS-related mortality (46). These findings consolidated the understanding that the tremendous volumetric increment with ALPPS may not translate into a coordinately enhanced recovery of liver function. Hepatobiliary scintigraphy studies have suggested that volumetry often overestimates liver function in ALPPS, whereas in PVE the function increase is even more pronounced than the volume increase (55, 56). One histological explanation is that the regenerative hepatocytes and biliary duct networks of rapidly grown livers in ALPPS are usually immature (57, 58). Collectively, these observations highlighted the necessity of concurrent functional assessment during the interstage course of ALPPS instead of overreliance on volumetric data.

Outside of concerns over its safety profiles, several initial studies have also reported extremely high rates of early postoperative tumor recurrence following ALPPS. In a single center study in 2013, six out of seven ALPPS patients experienced tumor recurrence over a median follow-up of 15 months (59). However, it should be noted that the mean number of CRLMs in this small case series reached 7.6, with a mean tumor diameter of 4.9 cm, suggesting a rather advanced stage of disease. It was previously speculated that accelerated hepatocellular hypertrophy may also essentially stimulate the growth of residual tumor cells. Nonetheless, both *in vivo* and *in vitro* studies have documented that ALPPS is not associated with the accelerated tumor growth in the FLR despite the enhanced regeneration process (60). Therefore, the tumor progression after ALPPS is most likely a reflection of the natural history of the disease itself.

In the setting of TSH, the waiting period between stages is generally 4–8 weeks, which is well-described as bearing the risk of tumor progression (7, 61). Dramatically shortening the waiting intervals, as in ALPPS, may however, not facilitate the assessment of interstage tumor growth and could consequently lead to impaired patient selections. In other words, compared with that in TSH, the manifestation of disease progression in ALPPS may shift from the waiting period to after stage 2. Consistent with the majority of recent comparative studies, the results of the present synthesis suggest that the two treatment modalities for CRLM had comparable short-term oncological benefits (62). Nonetheless, the paucity of data on long-term oncological outcomes hinders a further comparison between these two surgical procedures. This may also be inevitable due to the relative novelty of the surgeries.

From a clinical perspective, traditional TSH has been frequently regarded as a preferred treatment modality in earlier studies, and ALPPS was reserved as an alternative, typically after failed PVE or PVL. At present, this paradigm may have changed, as there is growing evidence indicating that the surgical benefits conferred by ALPPS are at least not inferior to those conferred by TSH according to both intention-to-treat and per protocol analyses (63). In light of both our and others' recent findings, whether TSH or ALPPS is optimal for patients with advanced CRLM depends on several factors other than the procedures themselves. That is, a careful evaluation of the patient characteristics is also of importance when balancing the benefits and risks, as well as the surgeons' experiences. For example, ALPPS may now be considered first by an experienced surgeon for those patients who present with critically limited FLR, extensive tumor burden and fairly good surgical tolerance. Nonetheless, determining the optimal selection of patients for ALPPS or TSH is beyond the scope of the current study and merits further investigations with larger cohorts and correction of center experience.

Although we herein focused on the management of CRLM with ALPPS, promising results have also been increasingly reported when testing ALPPS with primary liver malignancies, which are highly aggressive and generally arise from a background of cirrhosis or cholestasis (64, 65). Unfortunately, most of these results were derived from case series, with a scarcity of data on comparisons with traditional TSH. More studies in this field would lead the way to open up the surgical benefits of ALPPS to wider populations of patients with otherwise unresectable hepatobiliary malignancies.

There are several limitations to be acknowledged in the current meta-analysis. Six of the included studies were retrospective in nature, while there was only one RCT. Nevertheless, the majority of the included retrospective studies had relatively high quality, and the heterogeneity was acceptable on most of the outcome variables. Moreover, regarding the evidence level of the literature, meta-analyses of RCTs of a surgical procedure are actually not necessarily superior to those of contemporaneous non-RCTs (66). While we excluded comparative studies with indications of heterogeneity, these studies have also included a certain proportion of subjects diagnosed with CRLM, which may incur potential risks of selection bias. In addition, although the first stage of TSH typically involves the complete clearance of the FLR and contralateral PVE or PVL, not all of the TSH patients in the included studies had strictly local clearance of the FLR in the first stage. This otherwise simplified surgical procedure in the TSH group may have led to their morbidity and mortality rates being underestimated.

## Conclusions

The present meta-analysis confirmed that ALPPS for advanced CRLM achieves higher resectability than TSH but at the cost of increased rates of MiCs. The perioperative mortality, frequencies of major adverse events and short-term oncological outcomes of ALPPS have improved a lot than initial described, approaching a level similar to that of traditional TSH. Our study justified the clinical expansion of ALPPS in the management of initially unresectable CRLM with meticulous patient selection and gaining surgical experience.

## Data Availability Statement

The raw data supporting the conclusions of this article will be made available by the authors, without undue reservation.

## Author Contributions

LZ, ZY, and SZhe designed the study. The literature search, study selection, data extraction, and literature quality assessment were performed by ZY, SZha, and WW. SZhe made substantial contributions to acquisition, analysis, and interpretation of data. LZ wrote the manuscript. SZhe revised the manuscript critically for important intellectual content. All authors have read and approved the final manuscript.

## Conflict of Interest

The authors declare that the research was conducted in the absence of any commercial or financial relationships that could be construed as a potential conflict of interest.

## References

[1] Kron P Linecker M Jones RP Toogood GJ Clavien PA Lodge JPA Ablation or resection for colorectal liver metastases? A systematic review of the literature. Front Oncol. (2019) 9:1052 10.3389/fonc.2019.01052 31750233 PMC6843026

[2] Imai K Adam R Baba H. How to increase the resectability of initially unresectable colorectal liver metastases: a surgical perspective. Ann Gastroenterol Surg. (2019) 3:476–86. 10.1002/ags3.12276 31549007 PMC6749948

[3] Torzilli G Adam R Vigano L Imai K Goransky J Fontana A. Surgery of colorectal liver metastases: pushing the limits. Liver Cancer. (2016) 6:80–9. 10.1159/000449495 27995092 PMC5159716

[4] Treska V. Methods to increase future liver remnant volume in patients with primarily unresectable colorectal liver metastases: current state and future perspectives. Anticancer Res. (2016) 36:2065–71. 27127106

[5] Turrini O Ewald J Viret F Sarran A Goncalves A Delpero JR. Two-stage hepatectomy: who will not jump over the second hurdle? Eur J Surg Oncol. (2012) 38:266–73. 10.1016/j.ejso.2011.12.009 22244437

[6] Wicherts DA Miller R de Haas RJ Bitsakou G Vibert E Veilhan LA. Long-term results of two-stage hepatectomy for irresectable colorectal cancer liver metastases. Ann Surg. (2008) 248:994–1005. 10.1097/SLA.0b013e3181907fd9 19092344

[7] Brouquet A Abdalla EK Kopetz S Garrett CR Overman MJ Eng C. High survival rate after two-stage resection of advanced colorectal liver metastases: response-based selection and complete resection define outcome. J Clin Oncol. (2011) 29:1083–90. 10.1200/JCO.2010.32.6132 21263087 PMC3068054

[8] Narita M Oussoultzoglou E Jaeck D Fuchschuber P Rosso E Pessaux P. Two-stage hepatectomy for multiple bilobar colorectal liver metastases. Br J Surg. (2011) 98:1463–75. 10.1002/bjs.7580 21710481

[9] Schnitzbauer AA Lang SA Goessmann H Nadalin S Baumgart J Farkas SA. Right portal vein ligation combined with in situ splitting induces rapid left lateral liver lobe hypertrophy enabling 2-staged extended right hepatic resection in small-for-size settings. Ann Surg. (2012) 255:405–14. 10.1097/SLA.0b013e31824856f5 22330038

[10] Nadalin S Capobianco I Li J Girotti P Konigsrainer I Konigsrainer A. Indications and limits for associating liver partition and portal vein ligation for staged hepatectomy (ALPPS). Lessons learned from 15 cases at a single centre. Zeitschrift fur Gastroenterol. (2014) 52:35–42. 10.1055/s-0033-1356364 24420797

[11] Enne M Schadde E Bjornsson B Hernandez Alejandro R Steinbruck K Viana E. ALPPS as a salvage procedure after insufficient future liver remnant hypertrophy following portal vein occlusion. HPB. (2017) 19:1126–9. 10.1016/j.hpb.2017.08.013 28917644

[12] Tschuor C Croome KP Sergeant G Cano V Schadde E Ardiles V. Salvage parenchymal liver transection for patients with insufficient volume increase after portal vein occlusion – an extension of the ALPPS approach. Eur J Surg Oncol. (2013) 39:1230–5. 10.1016/j.ejso.2013.08.009 23994139

[13] Robles-Campos R Brusadin R López-Conesa A López-López V Navarro-Barrios Á López-Espín JJ. Long-term outcome after conventional two-stage hepatectomy versus tourniquet-ALPPS in colorectal liver metastases: a propensity score matching analysis. World J Surg. (2019) 43:2281–9. 10.1007/s00268-019-05031-w 31119359

[14] Adam R Imai K Benitez CC Allard MA Vibert E Cunha AS. Outcome after associating liver partition and portal vein ligation for staged hepatectomy and conventional two-stage hepatectomy for colorectal liver metastases. Br J Surg. (2016) 103:1521–9. 10.1002/bjs.10256 27517369

[15] Ratti F Schadde E Masetti M Massani M Zanello M Serenari M. Strategies to increase the resectability of patients with colorectal liver metastases: a multi-center case-match analysis of ALPPS and conventional two-stage hepatectomy. Ann Surg Oncol. (2015) 22:1933–42. 10.1016/j.hpb.2016.01.439 25564160

[16] Moris D Ronnekleiv-Kelly S Kostakis ID Tsilimigras DI Beal EW Papalampros A. Operative results and oncologic outcomes of associating liver partition and portal vein ligation for staged hepatectomy (ALPPS) versus two-stage hepatectomy (TSH) in patients with unresectable colorectal liver metastases: a systematic review and meta-analysis. World J Surg. (2018) 42:806–15. 10.1007/s00268-017-4181-6 28798996

[17] Shindoh J Vauthey JN Zimmitti G Curley SA Huang SY Mahvash A. Analysis of the efficacy of portal vein embolization for patients with extensive liver malignancy and very low future liver remnant volume, including a comparison with the associating liver partition with portal vein ligation for staged hepatectomy approach. J Am Coll Surg. (2013) 217:126–33; discussion 33–4. 10.1016/j.jamcollsurg.2013.03.004 23632095 PMC3880191

[18] Schadde E Ardiles V Slankamenac K Tschuor C Sergeant G Amacker N. ALPPS offers a better chance of complete resection in patients with primarily unresectable liver tumors compared with conventional-staged hepatectomies: results of a multicenter analysis. World J Surg. (2014) 38:1510–9. 10.1007/s00268-014-2513-3 24748319

[19] Sandstrom P Rosok BI Sparrelid E Larsen PN Larsson AL Lindell G. ALPPS improves resectability compared with conventional two-stage hepatectomy in patients with advanced colorectal liver metastasis results from a scandinavian multicenter randomized controlled trial (LIGRO trial). Ann Surg. (2018) 267:833–40. 10.1097/SLA.0000000000002511 28902669 PMC5916470

[20] Moher D Liberati A Tetzlaff J Altman DG PRISMA group T Preferred reporting items for systematic reviews and meta-analyses: the PRISMA statement. PLoS Med. (2009) 6:e1000097 10.1371/journal.pmed.1000097 19621072 PMC2707599

[21] Stang A. Critical evaluation of the newcastle-ottawa scale for the assessment of the quality of nonrandomized studies in meta-analyses. Eur J Epidemiol. (2010) 25:603–5. 10.1007/s10654-010-9491-z 20652370

[22] Deeks JJ Higgins J Altman DG Green S Cochrane handbook for systematic reviews of interventions version 5.1. 0 (updated March 2011). Cochrane Collaboration. (2011) 2:20011 Available online at: www.cochrane-handbook.org.

[23] Pan L Chen M Ji L Zheng L Yan P Fang J. The safety and efficacy of laparoscopic common bile duct exploration combined with cholecystectomy for the management of cholecysto-choledocholithiasis: an up-to-date meta-analysis. Ann Surg. (2018) 268:247–53. 10.1097/SLA.0000000000002731 29533266

[24] Laporte S Chapelle C Trone JC Bertoletti L Girard P Meyer G Early detection of the existence or absence of the treatment effect: a cumulative meta-analysis. J Clin Epidemiol. (2020) 124:24–33. 10.1016/j.jclinepi.2020.04.006 32298778

[25] Kambakamba P Linecker M Alvarez FA Samaras P Reiner CS Raptis DA. Short chemotherapy-free interval improves oncological outcome in patients undergoing two-stage hepatectomy for colorectal liver metastases. Ann Surg Oncol. (2016) 23:3915–23. 10.1245/s10434-016-5419-5 27431413

[26] Kikuchi Y Hiroshima Y Matsuo K Murakami T Kawaguchi D Endo I. Remnant liver tumor growth activity during treatment associating liver partition and portal vein occlusion for staged hepatectomy (ALPPS). J Gastrointest Surg. (2017) 21:1851–8. 10.1007/s11605-017-3523-x 28785935

[27] Baumgart J Jungmann F Bartsch F Kloth M Mittler J Heinrich S. Two-stage hepatectomy and alpps for advanced bilateral liver metastases: a tailored approach balancing risk and outcome. J Gastrointest Surg. (2019) 23:2391–400. 10.1007/s11605-019-04145-9 30820795

[28] Røsok BI Høst-Brunsell T Brudvik KW Carling U Dorenberg E Björnsson B. Characterization of early recurrences following liver resection by ALPPS and two stage hepatectomy in patients with colorectal liver-metastases and small future liver remnants; a translational substudy of the LIGRO-RCT. HPB. (2019) 21:1017–23. 10.1016/j.hpb.2018.06.008 30765198

[29] Jaeck D Oussoultzoglou E Rosso E Greget M Weber JC Bachellier P. A two-stage hepatectomy procedure combined with portal vein embolization to achieve curative resection for initially unresectable multiple and bilobar colorectal liver metastases. Ann Surg. (2004) 240:1037–49; discussion 49–51. 10.1097/01.sla.0000145965.86383.89 15570209 PMC1356519

[30] Eshmuminov D Raptis DA Linecker M Wirsching A Lesurtel M Clavien PA. Meta-analysis of associating liver partition with portal vein ligation and portal vein occlusion for two-stage hepatectomy. Br J Surg. (2016) 103:1768–82. 10.1002/bjs.10290 27633328

[31] Ratti F Cipriani F Gagliano A Catena M Paganelli M Aldrighetti L. Defining indications to ALPPS procedure: technical aspects and open issues. Updates Surg. (2014) 66:41–9. 10.1007/s13304-013-0243-y 24343420

[32] Linecker M Kambakamba P Raptis DA Malago M Ratti F Aldrighetti L. ALPPS in neuroendocrine liver metastases not amenable for conventional resection - lessons learned from an interim analysis of the International ALPPS Registry. HPB. (2020) 22:537–44. 10.1016/j.hpb.2019.08.011 31540885

[33] Linecker M Björnsson B Stavrou GA Oldhafer KJ Lurje G Neumann U. Risk adjustment in ALPPS is associated with a dramatic decrease in early mortality and morbidity. Ann Surg. (2017) 266:779–86. 10.1097/SLA.0000000000002446 28806301

[34] Schadde E Ardiles V Robles-Campos R Malago M Machado M Hernandez-Alejandro R. Early survival and safety of ALPPS first report of the international ALPPS registry. Ann Surg. (2014) 260:829–38. 10.1097/SLA.0000000000000947 25379854

[35] Xiang F Hu ZM. Chance and challenge of associating liver partition and portal vein ligation for staged hepatectomy. Hepatobiliary Pancreat Dis Int. (2019) 18:214–22. 10.1016/j.hbpd.2019.04.006 31056484

[36] Cao YL Wang GJ Li W. [Meta-analysis of the outcomes of associating liver partition and portal vein ligation for staged hepatectomy versus portal vein embolization for the treatment of liver cancer with insufficient future liver remnant]. Zhonghua Wai Ke Za Zhi. (2019) 57:540–8. 10.3760/cma.j.issn.0529-5815.2019.07.012 31269618

[37] Liu Y Yang Y Gu S Tang K A systematic review and meta-analysis of associating liver partition and portal vein ligation for staged hepatectomy (ALPPS) versus traditional staged hepatectomy. Medicine. (2019) 98:e15229 10.1097/MD.0000000000015229 30985727 PMC6485722

[38] Pandanaboyana S Bell R Hidalgo E Toogood G Prasad KR Bartlett A. A systematic review and meta-analysis of portal vein ligation versus portal vein embolization for elective liver resection. Surgery. (2015) 157:690–8. 10.1016/j.surg.2014.12.009 25704417

[39] Shen YN Guo CX Wang LY Pan Y Chen YW Bai XL. Associating liver partition and portal vein ligation versus 2-stage hepatectomy: a meta-analysis. Medicine. (2018) 97:e12082. 10.1097/MD.0000000000012082 30170426 PMC6392767

[40] Tustumi F Ernani L Coelho FF Bernardo WM Junior SS Kruger JAP. Preoperative strategies to improve resectability for hepatocellular carcinoma: a systematic review and meta-analysis. HPB. (2018) 20:1109–18. 10.1016/j.hpb.2018.06.1798 30057123

[41] Zhang Y Zheng Y Dong X Sun X Jiang X Wang Z. Associating liver partition and portal vein ligation for staged hepatectomy versus conventional staged hepatectomy: a meta-analysis. Minerva Med. (2018) 109:141–9. 10.23736/S0026-4806.17.05096-0 28398026

[42] Zhou Z Xu M Lin N Pan C Zhou B Zhong Y. Associating liver partition and portal vein ligation for staged hepatectomy versus conventional two-stage hepatectomy: a systematic review and meta-analysis. World J Surg Oncol. (2017) 15:227. 10.1186/s12957-017-1295-0 29258518 PMC5738171

[43] Truant S Scatton O Dokmak S Regimbeau JM Lucidi V Laurent A. Associating liver partition and portal vein ligation for staged hepatectomy (ALPPS): impact of the inter-stages course on morbi-mortality and implications for management. Eur J Surg Oncol. (2015) 41:674–82. 10.1016/j.ejso.2015.01.004 25630689

[44] Charnsangavej C Clary B Fong Y Grothey A Pawlik TM Choti MA. Selection of patients for resection of hepatic colorectal metastases: expert consensus statement. Ann Surg Oncol. (2006) 13:1261–8. 10.1245/s10434-006-9023-y 16947009

[45] Wanis KN Linecker M Madenci AL Muller PC Nussler N Brusadin R. Variation in complications and mortality following ALPPS at early-adopting centers. HPB. (2020). 10.1016/j.hpb.2020.04.009. [Epub ahead of print]. 32456975 PMC7680722

[46] Schadde E Raptis DA Schnitzbauer AA Ardiles V Tschuor C Lesurtel M. Prediction of mortality after ALPPS stage-1: an analysis of 320 patients from the International ALPPS registry. Ann Surg. (2015) 262:780–5; discussion 5–6. 10.1097/SLA.0000000000001450 26583666

[47] Hernandez-Alejandro R Bertens KA Pineda-Solis K Croome KP. Can we improve the morbidity and mortality associated with the associating liver partition with portal vein ligation for staged hepatectomy (ALPPS) procedure in the management of colorectal liver metastases? Surgery. (2015) 157:194–201. 10.1016/j.surg.2014.08.041 25282528

[48] Edmondson MJ Sodergren MH Pucher PH Darzi A Li J Petrowsky H. Variations and adaptations of associated liver partition and portal vein ligation for staged hepatectomy (ALPPS): many routes to the summit. Surgery. (2016) 159:1058–72. 10.1016/j.surg.2015.11.013 26747229

[49] Huiskens J Schadde E Lang H Malago M Petrowsky H de Santibañes E Avoiding postoperative mortality after ALPPS-development of a tumor-specific risk score for colorectal liver metastases. HPB. (2019) 21:898–905. 10.1016/j.hpb.2018.11.010 30611560

[50] Mor E Al-Kurd A Yaacov AB Aderka D Nissan A Ariche A. Surgical outcomes of two-stage hepatectomy for colorectal liver metastasis: comparison to a benchmark procedure. Hepatobiliary Surg Nutr. (2019) 8:29–36. 10.21037/hbsn.2018.12.02 30881963 PMC6383016

[51] Passot G Chun YS Kopetz SE Zorzi D Brudvik KW Kim BJ. Predictors of safety and efficacy of 2-stage hepatectomy for bilateral colorectal liver metastases. J Am Coll Surg. (2016) 223:99–108. 10.1016/j.jamcollsurg.2015.12.057 26968325 PMC4925205

[52] Schlegel A Lesurtel M Melloul E Limani P Tschuor C Graf R. ALPPS: from human to mice highlighting accelerated and novel mechanisms of liver regeneration. Ann Surg. (2014) 260:839–46; discussion 46–7. 10.1097/SLA.0000000000000949 25379855

[53] Lang H Baumgart J Mittler J. Associating liver partition and portal vein ligation for staged hepatectomy in the treatment of colorectal liver metastases: current scenario. Digest Surg. (2018) 35:294–302. 10.1159/000488097 29621745

[54] Borger P Schneider M Frick L Langiewicz M Sorokin M Buzdin A. Exploration of the transcriptional landscape of ALPPS reveals the pathways of accelerated liver regeneration. Front Oncol. (2019) 9:1206. 10.3389/fonc.2019.01206 31824837 PMC6882302

[55] Olthof PB Tomassini F Huespe PE Truant S Pruvot FR Troisi RI. Hepatobiliary scintigraphy to evaluate liver function in associating liver partition and portal vein ligation for staged hepatectomy: liver volume overestimates liver function. Surgery. (2017) 162:775–83. 10.1016/j.surg.2017.05.022 28732555

[56] de Graaf W van Lienden KP van den Esschert JW Bennink RJ van Gulik TM. Increase in future remnant liver function after preoperative portal vein embolization. Br J Surg. (2011) 98:825–34. 10.1002/bjs.7456 21484773

[57] Matsuo K Hiroshima Y Yamazaki K Kasahara K Kikuchi Y Kawaguchi D. Immaturity of bile canalicular-ductule networks in the future liver remnant while associating liver partition and portal vein occlusion for staged hepatectomy (ALPPS). Ann Surg Oncol. (2017) 24:2456–64. 10.1245/s10434-017-5922-3 28612126

[58] Matsuo K Murakami T Kawaguchi D Hiroshima Y Koda K Yamazaki K. Histologic features after surgery associating liver partition and portal vein ligation for staged hepatectomy versus those after hepatectomy with portal vein embolization. Surgery. (2016) 159:1289–98. 10.1016/j.surg.2015.12.004 26775576

[59] Oldhafer KJ Donati M Jenner RM Stang A Stavrou GA. ALPPS for patients with colorectal liver metastases: effective liver hypertrophy, but early tumor recurrence. World J Surg. (2014) 38:1504–9. 10.1007/s00268-013-2401-2 24326456

[60] Kambakamba P Linecker M Schneider M Reiner CS Nguyen-Kim TDL Limani P. Impact of associating liver partition and portal vein ligation for staged hepatectomy (ALPPS) on growth of colorectal liver metastases. Surgery. (2018) 163:311–7. 10.1016/j.surg.2017.10.036 29248180

[61] Fischer C Melstrom LG Arnaoutakis D Jarnagin W Brown K D'Angelica M. Chemotherapy after portal vein embolization to protect against tumor growth during liver hypertrophy before hepatectomy. JAMA Surg. (2013) 148:1103–8. 10.1001/jamasurg.2013.2126 24173207

[62] Hasselgren K Rosok BI Larsen PN Sparrelid E Lindell G Schultz NA ALPPS improves survival compared with TSH in patients affected of CRLM: survival analysis from the randomized controlled trial LIGRO. Ann Surg. (2019). 10.1097/SLA.0000000000003701. [Epub ahead of print]. 32049675

[63] Lang H de Santibañes E Schlitt HJ Malagó M van Gulik T Machado MA. 10th Anniversary of ALPPS - lessons learned and quo vadis. Ann Surg. (2019) 269:114–9. 10.1097/SLA.0000000000002797 29727331

[64] Chan A Zhang WY Chok K Dai J Ji R Kwan C ALPPS versus portal vein embolization for hepatitis-related hepatocellular carcinoma: a changing paradigm in modulation of future liver remnant before major hepatectomy. Ann Surg. (2019). 10.1097/SLA.0000000000003433. [Epub ahead of print]. 31305284

[65] Wang Z Peng Y Hu J Wang X Sun H Sun J. Associating liver partition and portal vein ligation for staged hepatectomy for unresectable Hepatitis B virus-related hepatocellular carcinoma: a single center study of 45 patients. Ann Surg. (2020) 271:534–41. 10.1097/SLA.0000000000002942 29995681

[66] Abraham NS Byrne CJ Young JM Solomon MJ. Meta-analysis of well-designed nonrandomized comparative studies of surgical procedures is as good as randomized controlled trials. J Clin Epidemiol. (2010) 63:238–45. 10.1016/j.jclinepi.2009.04.005 19716267

