# Supplementary material for: Conventional Two-Stage Hepatectomy or Associating Liver Partitioning and Portal Vein Ligation for Staged Hepatectomy for Colorectal Liver Metastases? A Systematic Review and Meta-Analysis
Source: Front Oncol. 2020 Aug 21;10:1391. doi: 10.3389/fonc.2020.01391 (PMC7471772; doi:10.3389/fonc.2020.01391)
Supplement: Supplementary file 15 [file Table_1.DOCX]

**Supplementary Table 1. Specific search strategies adapted for each electronic database.**

| Database | Search details |
| --- | --- |
| Pubmed  (https://www.ncbi.nlm.nih.gov/pubmed) | (((((((“colorectal liver metastasis”[Title/Abstract]) OR “liver metastases from colorectal cancer”[Title/Abstract]) OR CRLM[Title/Abstract])) OR ((((((Neoplasm Metastasis[MeSH Terms]) OR "Neoplasm Metastasis"[Title/Abstract]) OR metastasis[Title/Abstract])) AND ((((liver[MeSH Terms]) OR liver[Title/Abstract]) OR hepatic[Title/Abstract]) OR intrahepatic[Title/Abstract])) AND ((((Colorectal Neoplasms[MeSH Terms]) OR "Colorectal Neoplasms"[Title/Abstract]) OR "Colorectal cancer"[Title/Abstract]) OR "Colorectal Carcinoma"[Title/Abstract])))) AND (((((("liver partition"[Title/Abstract]) OR "in situ split liver resection"[Title/Abstract]) OR "in situ splitting"[Title/Abstract]) OR ("associating liver partition[Title/Abstract] AND portal vein ligation for staged hepatectomy"[Title/Abstract])) OR ("associated liver partition[Title/Abstract] AND portal vein ligation for staged hepatectomy"[Title/Abstract])) OR ALPPS[Title/Abstract])) AND ((((((hepatectomy[MeSH Terms]) OR hepatectomy[Title/Abstract]) OR "liver resection"[Title/Abstract]) OR two stage hepatectomy[Title/Abstract]) OR TSH[Title/Abstract]) OR "two stage liver resection"[Title/Abstract]) |
| Web of science  (https://apps.webofknowledge.com) | #1 TOPIC: (colorectal liver metastasis) OR TOPIC: (liver metastases from colorectal cancer) OR TOPIC: (CRLM) OR TOPIC: (CLM)  #2 TOPIC: (liver partition) OR TOPIC: (in situ split liver resection) OR TOPIC: (in situ splitting) OR TOPIC: (associating liver partition and portal vein ligation for staged hepatectomy) OR TOPIC: (associated liver partition and portal vein ligation for staged hepatectomy) OR TOPIC: (ALPPS)  #3 TOPIC: (two stage hepatectomy) OR TOPIC: (staged hepatectomy) OR TOPIC: (TSH) OR TOPIC: (two stage liver resection)  #3 AND #2 AND #1 |
| Embase  (https://www.embase.com) | #1 'colorectal liver metastasis'/exp  #2 'colorectal liver metastasis':ti,ab,kw OR 'liver metastases from colorectal cancer':ti,ab,kw OR crlm:ti,ab,kw OR clm:ti,ab,kw  #3 #1 OR #2  #4 'associating liver partition and portal vein ligation for staged hepatectomy'/exp  #5 'associating liver partition':ti,ab,kw AND 'portal vein ligation for staged hepatectomy':ti,ab,kw OR 'liver partition':ti,ab,kw OR 'in situ split liver resection':ti,ab,kw OR 'in situ splitting':ti,ab,kw OR ('associated liver partition':ti,ab,kw AND 'portal vein ligation for staged hepatectomy':ti,ab,kw) OR alpps:ti,ab,kw  #6 #4 OR #5  #7 'liver resection'/exp  #8 'liver resection':ti,ab,kw OR hepatectomy:ti,ab,kw OR 'two stage hepatectomy':ti,ab,kw OR tsh:ti,ab,kw OR 'staged hepatectomy':ti,ab,kw OR 'two stage resection':ti,ab,kw  #9 #7 OR #8  #10 #9 AND #6 AND #3 |
| Cochrane library  (https://www.cochranelibrary.com) | #1 (colorectal liver metastasis):ti,ab,kw OR (liver metastases from colorectal cancer):ti,ab,kw OR (CLM):ti,ab,kw OR (CRLM):ti,ab,kw  #2 (associating liver partition and portal vein ligation for staged hepatectomy):ti,ab,kw OR (associated liver partition and portal vein ligation for staged hepatectomy):ti,ab,kw OR (ALPPS):ti,ab,kw OR (liver partition):ti,ab,kw OR (in-situ split liver resection):ti,ab,kw  #3 MeSH descriptor: [Hepatectomy] explode all trees  #4 (hepatectomy):ti,ab,kw OR (two stage hepatectomy):ti,ab,kw OR (two stage liver resection):ti,ab,kw OR (TSH):ti,ab,kw OR (staged hepatectomy):ti,ab,kw  #5 #3 OR #4  #6 #1 AND #2 AND #5 |
