# Supplementary material for: Conventional Two-Stage Hepatectomy or Associating Liver Partitioning and Portal Vein Ligation for Staged Hepatectomy for Colorectal Liver Metastases? A Systematic Review and Meta-Analysis
Source: Front Oncol. 2020 Aug 21;10:1391. doi: 10.3389/fonc.2020.01391 (PMC7471772; doi:10.3389/fonc.2020.01391)
Supplement: Supplementary file 16 [file Table_2.DOCX]

**Supplementary Table 2. Summary of demographic characteristics of patients from the included studies.**

| Author  (year) | Observation period | No. of patients | Age  (year) | Gender  (male, %) | ASA score | | Associated comorbidities | Primary tumor location | |
| --- | --- | --- | --- | --- | --- | --- | --- | --- | --- |
|  |  |  |  |  | I-II | III-IV |  | Colon | Rectum |
| Robles-Campos *et al* (2019) | 2011-2016 /  2000-2011 | 21/21 | 66 (44–83)/  59 (47–74) | 15 (71.4%) /  14 (66.7%) | 12 (57.1%) /  6 (28.6%) | 9 (42.9%) /  15 (71.4%) | ND | 14 (67%) /  16 (76%) | 7 (33%) /  5 (24%) |
| Baumgart *et al* (2019) | 2008-2017 /  2008-2017 | 8/50 | ND | 4 (50%)/  36 (72%) | ND | ND | ND | 4 (50%) /  34 (68%) | 4 (50%) /  16 (32%) |
| Sandstrom *et al* (2018) | 2014-2016 /  2014-2016 | 48/49 | 65.4 ± 8.9 /  64.9 ± 11.7 | 32 (66.7%) /  36 (73.5%) | 44 (91.7%) / 40 (81.6%) | 4 (8.3%) /  9 (18.4%) | ND | 28 (58%) /  29 (59%) | 20 (42%) /  20 (41%) |
| Rosok *et al*  (2018) | 2014-2016 /  2014-2016 | 13/11 | ND | ND | ND | ND | ND | ND | |
| Kikuchi *et al*  (2017) | By April 2015/  By April 2015 | 12/20 | 66 ± 2.5 /  62.5 ± 2.7 | 6 (50%) /  12 (60%) | ND | ND | ND | ND | |
| Kambakamba *et al* (2016) | 2011-2015 /  2008-2015 | 43/31 | ND | ND | ND | ND | ND | ND | |
| Adam *et al*  (2016) | 2010-2014 /  2010-2014 | 17/41 | 58 (23–75) /  58 (32–75) | 12 (70.6%) /  23 (56.1%) | 13 (76.5%) / 37 (90.3%) | 4 (23.5%)/  4 (9.8%) | ND | 13 (76%) /  27 (66%) | 4 (24%) /  14 (34%) |
| Ratti *et al*  (2015) | 2012-2013 /  2008-2013 | 12/36 | 59 (51–79) /  59 (42–66) | 5 (41.7%) /  19 (52.8%) | 9 (75%) /  26 (72.3%) | 3 (25%) /  10 (27.8%) | 4 (33.3%) /  16 (44.4%) | 9 (75%) /  25 (69%) | 3 (25%) /  11 (31%) |

**Supplementary Table 2. Continued.**

| Author  (year) | Synchronous/metachronous | | Extrahepatic disease | CEA  (ng/ml) | No. of liver lesions | Tumor size  (mm) | NC | NC cycles |
| --- | --- | --- | --- | --- | --- | --- | --- | --- |
|  | Synchronous | Metachronous |  |  |  |  |  |  |
| Robles-Campos *et al* (2019) | 15 (71%) /  17 (81%) | 6 (29%) /  4 (19%) | 5 (23.8%) /  4 (19%) | 18.5 (1–256) /  18 (1–285) | ND | 23 (20 - 50) /  30 (10 - 60) **^*^** | 19 (91%) /  21 (100%) | ND |
| Baumgart *et al* (2019) | 6 (75%)/  42 (84%) | 2 (25%) /  8 (16%) | 0 (0%) /  5 (10%) | ND | ND | ND | 7 (88%) /  46 (92%) | ND |
| Sandstrom *et al* (2018) | ND | | 9 (18.8%) /  7 (14.3%) | ND | 8 ± 4 /  8 ± 5 | 54 ± 41 /  49 ± 39**^#^** | 47 (98%) /  48 (98%) | 6 ± 4 /  7 ± 4 |
| Rosok *et al*  (2018) | ND | | ND | ND | ND | ND | ND | ND |
| Kikuchi *et al*  (2017) | 12 (100%) /  20 (100%) | 0 (0%)/  0 (0%) | 2 (16.6%) /  4 (20.0%) | 254 ± 213 /  81.1 ± 29.4 | 12 ± 1.4 /  15 ± 2.3 | ND | 12 (100%) /  20 (100%) | 18.0 ± 3.7 /  12.1 ± 2.1 |
| Kambakamba *et al* (2016) | ND | | ND | ND | ND | ND | 39 (91%) /  28 (90%) | 6 (5-8) /  6 (4-7) |
| Adam *et al*  (2016) | 15 (88%) /  38 (93%) | 2 (12%)/  3 (7%) | 6 (35.3%) /  12 (29.3%) | 8 (1–1195) / 7.9 (0.5–940) | 10 (3–20) /  10 (2–35) | 38 (8–140) /  43 (10–140) **^*^** | 17 (100%) /  41 (100%) | 8 (4–37) /  11 (4–32) |
| Ratti *et al*  (2015) | 5 (42%)/  13 (36%) | 7 (58%)/  23 (64%) | ND | ND | 4 (1–11) /  5 (1–13) | 44 ± 17.6 /  38 ± 25 **^#^** | 9 (75%) /  30 (83%) | 6 (2–12) /  6 (4–9) |

**^*^**Mean maximal diameter of the largest liver lesion;

**^#^**Median diameter of liver lesion;

Data was expressed as (ALPPS)/(TSH) in most blank, and described as mean ± SD, median (IQR), or number (percentage), depending on their presentation in the original publications; Abbreviations: *ASA*, American society of anesthesiology; *CEA*, carcinoembryonic antigen; *NC*, Neoadjuvant chemotherapy; *ND*, not described.
