# Supplementary material for: Conventional Two-Stage Hepatectomy or Associating Liver Partitioning and Portal Vein Ligation for Staged Hepatectomy for Colorectal Liver Metastases? A Systematic Review and Meta-Analysis
Source: Front Oncol. 2020 Aug 21;10:1391. doi: 10.3389/fonc.2020.01391 (PMC7471772; doi:10.3389/fonc.2020.01391)
Supplement: Supplementary file 17 [file Table_3.DOCX]

**Supplementary Table 3.** **Comparison of demographic characteristics of ALPPS versus TSH patients from the included studies.**

| Variables | No. of  studies | No. of  patients | Single armed synthesis | | OR/WMD  (95% CI) | *P* value | Heterogeneity  (I^2^, *p* value) |
| --- | --- | --- | --- | --- | --- | --- | --- |
|  |  |  | ALPPS group (95% CI) | TSH group (95% CI) |  |  |  |
| Age (years) | 2 | 129 | 65.86 [64.62, 67.09] | 62.78 [61.67, 63.89] | 3.00 [1.32, 4.69] | <0.001 | 40.8%, 0.19 |
| Gender (male) | 6 | 335 | 0.63 [0.54, 0.72] | 0.65 [0.58, 0.71] | 0.85 [0.52, 1.38] | 0.50 | 0%, 0.64 |
| ASA score (I-II) | 4 | 245 | 0.77 [0.58, 0.93] | 0.71 [0.47, 0.90] | 1.57 [0.80, 3.05] | 0.19 | 47.4%, 0.13 |
| Primary tumor  location (Colon) | 5 | 303 | 0.55 [0.36, 0.73] | 0.61 [0.47, 0.74] | 0.95 [0.57, 1.61] | 0.86 | 0%, 0.71 |
| Synchronous metastasis | 5 | 238 | 0.79 [0.56, 0.95] | 0.82 [0.58, 0.98] | 0.77 [0.35, 1.68] | 0.51 | 0%, 0.84 |
| Extrahepatic disease | 5 | 287 | 0.19 [0.12, 0.28] | 0.17 [0.12, 0.23] | 1.19 [0.63, 2.25] | 0.59 | 0%, 0.96 |
| No. of liver lesions | 2 | 129 | 10.02[ 6.10, 13.94] | 11.52 [4.66, 18.38] | -1.57 [-4.51, 1.37] | 0.30 | 85.9%, 0.01 |
| Mean tumor diameter  (mm) | 2 | 129 | 48.24 [40.69, 55.80] | 42.88 [32.17, 53.59] | 5.61 [-4.41, 15.62] | 0.27 | 0%, 0.92 |
| NC | 7 | 439 | 0.95 [0.91, 0.99] | 0.96 [0.91, 1.00] | 0.66 [0.27, 1.61] | 0.36 | 0%, 0.9 |
| NC cycles | 2 | 129 | 11.97 [0.21, 23.73] | 9.56 [4.56, 14.56] | 2.40 [-4.36, 9.16] | 0.49 | 95.8%, 0.001 |

Abbreviations: *ALPPS,* associating liver partitioning and portal vein ligation for staged hepatectomy; *ASA*, American society of anesthesiology; *CI,* confidence interval; *NC*, neoadjuvant chemotherapy; *OR*, odds ratio; *TSH*, two-stage hepatectomy; WMD, weighted mean difference.
