# Supplementary material for: Conventional Two-Stage Hepatectomy or Associating Liver Partitioning and Portal Vein Ligation for Staged Hepatectomy for Colorectal Liver Metastases? A Systematic Review and Meta-Analysis
Source: Front Oncol. 2020 Aug 21;10:1391. doi: 10.3389/fonc.2020.01391 (PMC7471772; doi:10.3389/fonc.2020.01391)
Supplement: Supplementary file 18 [file Table_4.DOCX]

**Supplementary Table 4. Summary of previous meta-analyses on comparisons between ALPPS and other** **FLR augmentation strategies.**

| **Author (year)** | **Included studies** | **Comparisons** | **Surgical indications** | **No. of patients** | **Main findings** |
| --- | --- | --- | --- | --- | --- |
| Pandanaboyana et al  (2015) | 2 retrospective studies | ALPPS vs PVE | Patients requiring staged hepatectomy | N=191 (ALPPS, 32; PVE, 159) | ALPPS resulted in increased hypertrophy of the FLR than PVE. |
| Eshmuminov et al  (2016) | 9 retrospective studies | ALPPS vs  PVE/PVL+PVE vs PVL | Patients requiring staged hepatectomy | N=357 (ALPPS, 55; PVE, 179; PVL, 123) | ALPPS resulted in increased FLR hypertrophy and RR, while had higher morbidity and mortality rates than PVE. |
| Zhang et al  (2017) | 7 retrospective studies | ALPPS vs TSH | Patients requiring staged hepatectomy | N=525  (ALPPS, 126;  TSH, 399) | ALPPS resulted in higher RR and FLR hypertrophy ratio while had increased rate of MaCs than TSH; ALPPS and TSH were similar in MiCs and PHLF, morbidity and mortality rates. |
| Zhou et al  (2017) | 9 retrospective studies  + 1 RCT | ALPPS vs TSH | Primary or secondary liver tumor requiring staged hepatectomy | N=719 (ALPPS, 201; PVE, 518) | ALPPS had faster FLR hypertrophy, higher RR and R0 rate, shortened waiting interval while similar 90-day mortality rate relative to TSH. |
| Moris et al  (2018) | 9 retrospective studies | ALPPS vs TSH | Unresectable CRLM | N=657 (ALPPS, 186; TSH, 471) | Despite similar survival rates, ALPPS resulted in faster FLR growth but higher morbidity and mortality rates than TSH. |
| Shen et al  (2018) | 7 retrospective studies | ALPPS vs TSH | Patients requiring staged hepatectomy | N=561 (ALPPS, 136; TSH, 425) | ALPPS had higher RR relative to TSH, while the two groups were similar in PHLF and perioperative morbidity and mortality rates. |
| Tustumi et al  (2018) | 45 retrospective studies | PVE/PVL  vs TACE + PVE  vs radioembolization  vs ALPPS | Hepatocellular carcinoma | N=1284  (ALPPS, 161) | ALPPS had higher PHLF and 90d mortality rates than PVE/PVL and TACE + PVE; ALPPS and radioembolization showed increased rates of MaCs than PVE/PVL and TACE + PVE. |
| Cao et al  (2019) | 9 cohort studies  + 1 RCT | ALPPS vs PVE | Liver cancer with insufficient FLR | N=620 (ALPPS, 165; PVE, 455) | ALPPS achieved higher RR, R0 rate, FLR growth rate and shorten waiting interval, while had similar morbidity and mortality rates than PVE. |
| Liu et al  (2019) | 8 retrospective studies  + 1 RCT | ALPPS vs  PVE/TSH | Liver cancer requiring extended liver resection | N=557 (ALPPS, 170; TSH, 180; PVE, 207) | ALPPS achieved greater FLR hypertrophy and RR, but resulted in greater morbidity and mortality. |

Abbreviations: *ALPPS,* associating liver partitioning and portal vein ligation for staged hepatectomy; *FLR*, future liver remnant; *MaCs,* major complications; *MiCs,* minor complications; *PHLF,* posthepatectomy liver failure; *PVE,* portal vein embolization; *PVL,* portal vein ligation; *TSH*, two-stage hepatectomy; *RCT,* randomized controlled trial; *RR,* resection rates; *TACE,* transcatheter arterial chemoembolization.
