# Supplementary material for: Conventional Two-Stage Hepatectomy or Associating Liver Partitioning and Portal Vein Ligation for Staged Hepatectomy for Colorectal Liver Metastases? A Systematic Review and Meta-Analysis
Source: Front Oncol. 2020 Aug 21;10:1391. doi: 10.3389/fonc.2020.01391 (PMC7471772; doi:10.3389/fonc.2020.01391)
Supplement: Supplementary file 19 [file Data_Sheet_1.docx]

**Supplementary Figure Legend**

**Supplementary Figure 1.** Quality Assessment of the included studies. (A). Quality score of the retrospective studies, according to the modified Newcastle-Ottawa Scale; (B). Quality assessment of randomized controlled trials, according to the Cochrane handbook for systematic reviews of interventions.

**Supplementary Figure 2.** Forest plots of 90-day mortality and major complications. (A). Forest plots of 90-day mortality; (B). Cumulative meta-analysis of 90-day mortality; (C) Forest plots of overall major complications (MaCs); (D). Cumulative meta-analysis of overall MaCs; E) Forest plots of stage 1 MaCs; E). Forest plots of stage 2 MaCs.

**Supplementary Figure 3.** Forest plots of minor complications, resection rate and R0 rate. Forest plots of overall minor complications (MiCs) (A), stage 1 MiCs(B), stage 2 MiCs (C), R0 rate (D) and resection rate (E). Cumulative meta-analysis of resection rate (F).

**Supplementary Figure 4.** Forest plots of blood loss, length of hospital stay and future liver remnant.

Forest plots of the amount of estimated blood loss during stage 1 (A) and stage 2 (B), length of hospital stay (C), waiting interval (D), preoperative future liver remnant (FLR) (E), preoperative FLR/total liver volume (TLV) (F), and FLR (G) and FLR/TLV ratio (H) at 1 week after first interventions.

**Supplementary Figure 5.** Forest plots of short-term oncological outcomes. (A). 1-year overall survival; (B). 1-year tumor recurrence; (C).1-year disease free survival.

**Supplementary Figure 6.** Forest plots of post-hepatectomy liver failure and biliary leakage. Forest plots of overall post-hepatectomy liver failure (PHLF) (A), stage 1 PHLF (B), stage 2 PHLF (C), overall biliary leakage (D), stage 1 biliary leakage (E) and stage 2 biliary leakage (F). Cumulative meta-analysis of overall PHLF (G) and stage 2 PHLF (H).

**Supplementary Figure 7.** L'Abbe plots of mortality, morbidity and surgical resectability. (A). 90-day mortality; (B). Overall major complications (MaCs); (C). Stage 1 MaCs; (D). Stage 2 MaCs; (E). Overall minor complications (MiCs); (F). Stage 1 MiCs; (G). Stage 2 MiCs; (H). R0 rate; (I). Resection rate.

**Supplementary Figure 8.** L'Abbe plots of short-term oncological outcomes, post-hepatectomy liver failure and biliary leakage. (A). 1-year overall survival; (B). 1-year tumor recurrence; (C). 1-year disease free survival. (D). Overall post-hepatectomy liver failure (PHLF); (E). Stage 1 PHLF; (F). Stage 2 PHLF; (G). Overall biliary leakage; (H). Stage 1 biliary leakage; (I). Stage 2 biliary leakage.

**Supplementary Figure 9.** Galbraith radial plots of mortality, morbidity and surgical resectability. (A). 90-day mortality; (B). Overall major complications (MaCs); (C). Stage 1 MaCs; (D). Stage 2 MaCs; (E). Overall minor complications (MiCs); (F). Stage 1 MiCs; (G). Stage 2 MiCs; (H). R0 rate; (I). Resection rate.

**Supplementary Figure 10.** Galbraith radial plots of short-term oncological outcomes, post-hepatectomy liver failure and biliary leakage. (A). 1-year overall survival; (B). 1-year tumor recurrence; (C). 1-year disease free survival. (D). Overall post-hepatectomy liver failure (PHLF); (E). Stage 1 PHLF; (F). Stage 2 PHLF; (G). Overall biliary leakage; (H). Stage 1 biliary leakage; (I). Stage 2 biliary leakage.

**Supplementary Figure 11.** Funnel plots of mortality, morbidity and surgical resectability. (A). 90-day mortality; (B). Overall major complications (MaCs); (C). Stage 1 MaCs; (D). Stage 2 MaCs; (E). Overall minor complications (MiCs); (F). Stage 1 MiCs; (G). Stage 2 MiCs; (H). R0 rate; (I). resection rate; (J). Quantitative evaluation for publication bias.

**Supplementary Figure 12.** Funnel plots of short-term oncological outcomes, post-hepatectomy liver failure and biliary leakage. (A). 1-year overall survival; (B). 1-year tumor recurrence; (C). 1-year disease free survival. (D). Overall post-hepatectomy liver failure (PHLF); (E). Stage 1 PHLF; (F). Stage 2 PHLF; (G). Overall biliary leakage; (H). Stage 1 biliary leakage; (I). Stage 2 biliary leakage; (J). Quantitative evaluation for publication bias.

**Supplementary Figure 13.** Sensitivity analyses of mortality, morbidity and surgical resectability. (A). 90-day mortality; (B). Overall major complications (MaCs); (C). Stage 1 MaCs; (D). Stage 2 MaCs; (E). Overall minor complications (MiCs); (F). Stage 1 MiCs; (G). Stage 2 MiCs; (H). R0 rate; (I). Resection rate.

**Supplementary Figure 14.** Sensitivity analyses of short-term oncological outcomes, post-hepatectomy liver failure and biliary leakage. (A). 1-year overall survival; (B). 1-year tumor recurrence; (C). 1-year disease free survival. (D). Overall post-hepatectomy liver failure (PHLF); (E). Stage 1 PHLF; (F). Stage 2 PHLF; (G). Overall biliary leakage; (H). Stage 1 biliary leakage; (I). Stage 2 biliary leakage.
